# Sailing in Dark Waters: The Challenging Management of Recurrent In-Stent Calcified Nodule Protrusion

**DOI:** 10.1016/j.jscai.2024.102457

**Published:** 2025-01-14

**Authors:** Daniel Bandeira, Steven Pfau, Daniel Chamié

**Affiliations:** a Section of Cardiovascular Medicine, Department of Internal Medicine, Yale School of Medicine, New Haven, Connecticut; b Cardiology Department, West Haven VA Medical Center, West Haven, Connecticut

**Keywords:** brachytherapy, calcium, in-stent restenosis, optical coherence tomography

## Case report

The patient was a 74-year-old man with hypertension, hypercholesterolemia, diabetes mellitus, prior myocardial infarction at 42 years of age, prior Hodgkin lymphoma, and treatment for prostate cancer. He had a long history of coronary revascularizations dating back to March and June 1999, with percutaneous coronary interventions (PCIs) to the left anterior descending artery (LAD). A LAD in-stent restenosis (ISR) led to coronary artery bypass grafting later that year, with a left internal mammary artery graft to the LAD and a saphenous vein graft to the right coronary artery. Additional nonspecified PCIs were performed 6, 7, and 10 years after the initial presentation. Twenty years later, in 2019, he underwent PCI with drug-eluting stent (DES) implantation in the native right posterior descending artery and saphenous vein graft to the right coronary artery. Three months later, an ISR of the posterior descending artery stent was redilated but was too distal for brachytherapy. In 2021, ISR of a left circumflex (LCx) stent was treated with another DES implantation extending to the LCx ostium.

**Figure 1:**
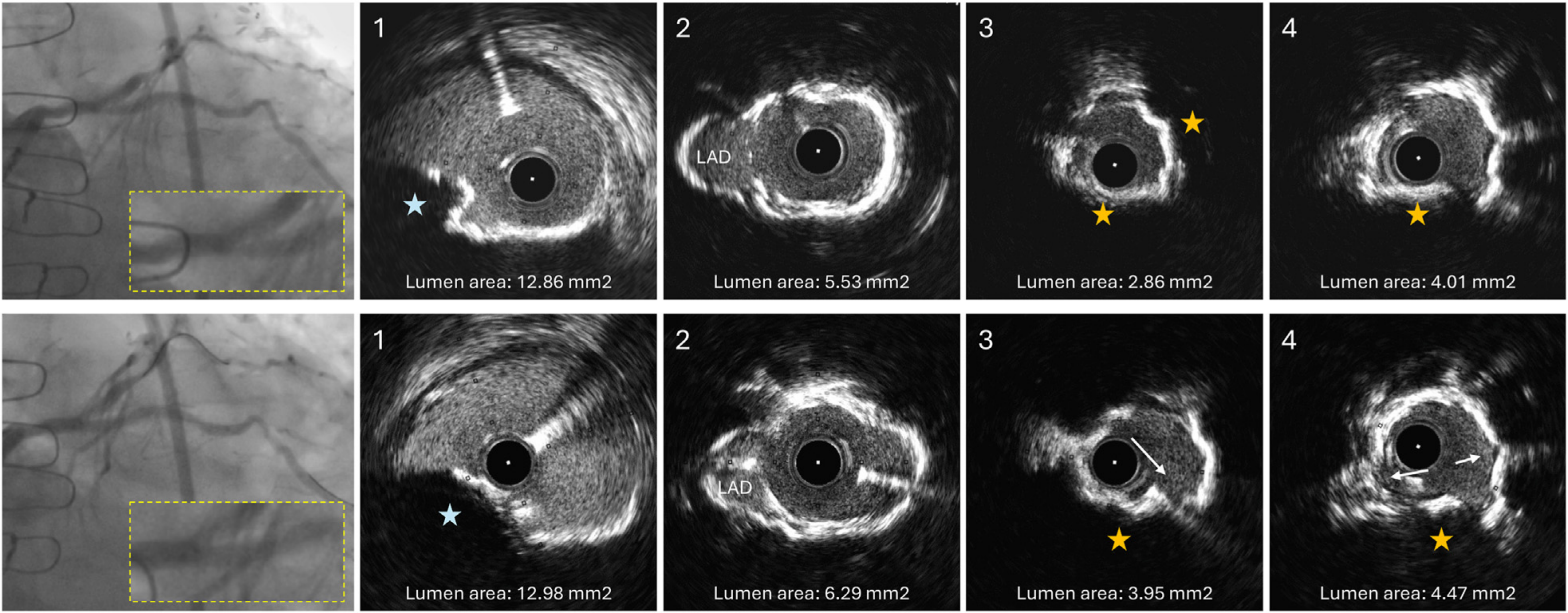
**IVUS images of the second in-stent restenosis at the LCx ostium (October 2023).** The top row presents the angiographic image of the LCx before intervention, with corresponding IVUS cross-sections acquired post-ELCA (the IVUS catheter did not cross the lesion initially). IVUS cross-sections are displayed from proximal (left) to distal (right) at the distal LMCA (1), LMCA bifurcation (2), and LCx ostium (3 and 4). A calcified nodule is evident in the LMCA (blue star in panel 1) without compromising the LMCA lumen area. The LCx stent extends up to the LMCA bifurcation with struts overlying the LAD ostium (panel 2). The first 5 mm of the LCx ostium is affected by significant in-stent calcification with calcified nodule protrusion between the stent struts into the lumen (yellow stars in panels 3 and 4). The MLA measured 2.86 mm^2^ (panel 3), and it was expected to be smaller before the ELCA application. The bottom row presents the angiography at the end of the procedure and IVUS cross-sections obtained after delivery of 80 pulses of intravascular lithotripsy and dilatations with 3.0-mm and 3.5-mm noncompliant balloons. The IVUS cross-sections are presented from proximal (left) to distal (right) matching the locations of the previous IVUS run. The white arrows identify calcium fractures. A lumen gain of 1.0 mm^2^ was obtained at the MLA site. The inset in the angiographies provides a magnified view of the region of interest involving the distal LMCA and proximal LCx stent. ELCA, excimer laser coronary atherectomy; IVUS, intravascular ultrasound; LAD, left anterior descending artery; LCx, left circumflex artery; LMCA, left main coronary artery; MLA, minimum lumen area.

Two years after the first LCx restenosis, in October 2023, another coronary angiography performed due to stable angina revealed a recurrent focal ISR at the LCx ostium (Figure 1 [top row] and Supplemental Video 1). Excimer laser coronary atherectomy was performed followed by intravascular ultrasound, which showed 2 layers of stents covered with minimal neointimal hyperplasia, diffuse calcified neoatherosclerosis, and a calcified nodule (CN) protruding between the stents into the lumen in the LCx ostium (Figure 1, top panel and Supplemental Video 2). The minimum lumen area measured 2.86 mm^2^ at the CN protrusion site. A nonobstructive CN was also present in the distal unstented left main coronary artery (LMCA) (lumen area: 12.86 mm^2^). Intravascular lithotripsy and dilatations with 3.0-mm and 3.5-mm noncompliant balloons were applied to the LCx ostium, promoting calcium fracture and a lumen gain of 1.09 mm^2^ (Figure 1 [bottom row] and Supplemental Video 2). The procedure was completed with brachytherapy.

**Figure 2:**
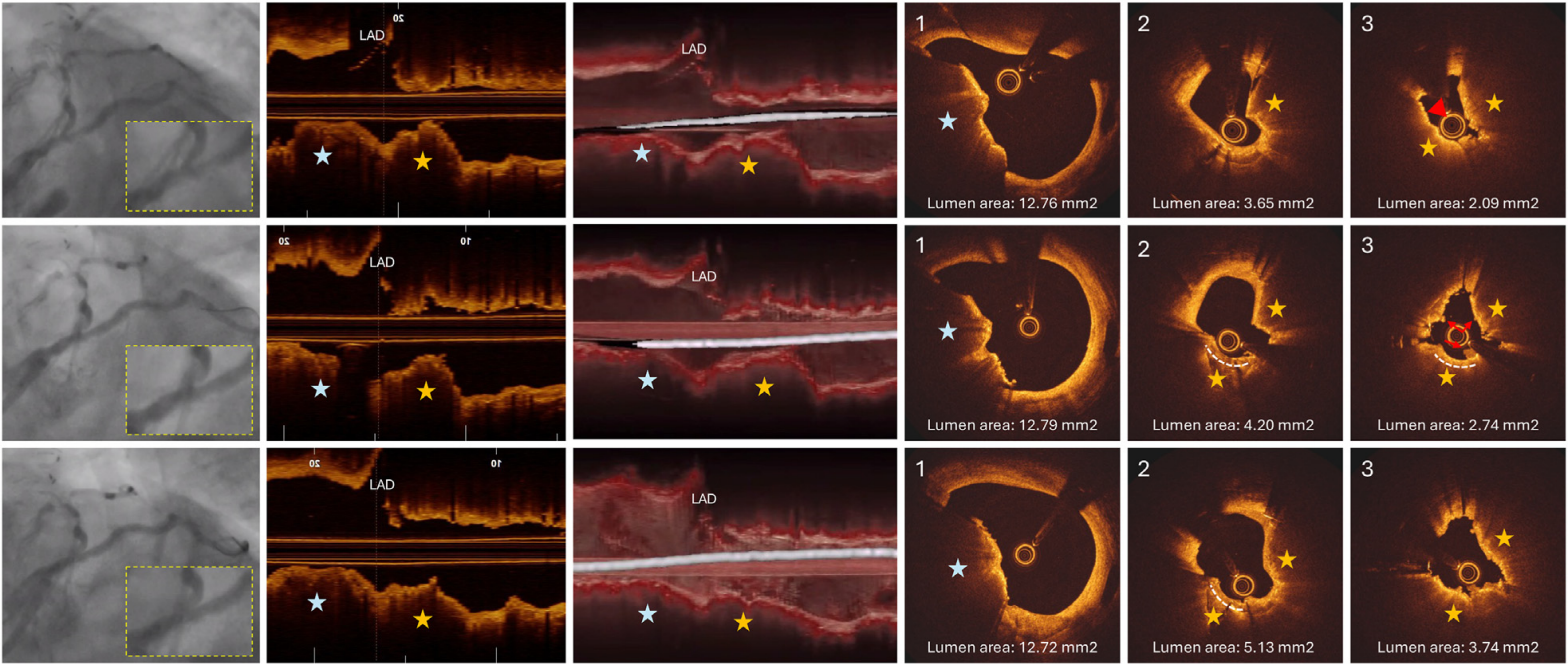
**OCT images of the third in-stent restenosis at the LCx ostium (August 2024).** The top row presents the angiographic image preintervention, a longitudinal OCT view, a 3D longitudinal vessel cutaway OCT reconstruction, and OCT cross-sections at the LMCA (image 1) and LCx ostium (images 2 and 3). Due to the inability to initially cross the lesion with the OCT catheter, this OCT run was acquired after predilatation with a 2.0-mm semicompliant balloon. A CN reprotrusion through a dual layer of stents is visible within the 5-mm segment of the LCx ostium (yellow stars) promoting reobstruction of the lumen (MLA postdilatation with 2.0-mm balloon: 2.09 mm^2^; image 3). The red arrowhead in image 3 indicates an irregular lumen surface, which may represent thrombus superimposed on the CN surface. The CN located at the LMCA remained stable without compromising the LMCA lumen since the last procedure. The 3D longitudinal OCT view indicates that the guide wire was not in contact with the CN protrusion, influencing the decision to proceed with OA instead of rotational atherectomy for the CN debulking. The middle row presents the angiography and OCT images after OA. Debulking created by the OA crown is demonstrated by the dashed curved white line. Red arrows indicate small white thrombi. Small lumen gain is observed. The bottom row presents the final angiography after delivery of 120 pulses of intravascular lithotripsy and dilatations with 3.0-mm and 3.5-mm noncompliant balloons. Compression of the CN nodule is better evident in the longitudinal and 3D views, resulting in a final MLA of 3.74 mm^2^ and a lumen gain of 1.65 mm^2^. The inset in the angiographies provides a magnified view of the region of interest involving the distal LMCA and proximal LCx stent. 3D, 3-dimensional; CN, calcified nodule; LAD, left anterior descending artery; LCx, left circumflex artery; LMCA, left main coronary artery; MLA, minimum lumen area; OA, orbital atherectomy; OCT, optical coherence tomography.

Ten months later, in August 2024, the patient presented with a non–ST-elevation myocardial infarction. Angiography revealed a recurrence of a focal ISR at the LCx ostium (Figure 2 [top row] and Supplemental Video 3). Optical coherence tomography (OCT) images acquired after predilatation with a 2.0-mm semicompliant balloon revealed reprotrusion of the CN inside the stents, with a minimum lumen area of 2.09 mm^2^ (Figure 2 [top row] and Supplemental Video 4). The CN located in the LMCA was unchanged since the last procedure with preserved lumen dimensions. Due to suboptimal guide wire bias toward the CN after the lesion predilatation (Figure 2, 3-dimensional [3D] OCT in the top row), we chose orbital atherectomy, which promoted calcium debulking and superficial fractures with small lumen gain (Figure 2 [middle row] and Supplemental Video 4). Intravascular lithotripsy with a 3.0-mm balloon followed by high-pressure dilatations with 3.0-mm and 3.5-mm noncompliant balloons were performed at the LCx ostium, resulting in expansion of the CN region and a lumen gain of 1.65 mm^2^ (Figure 2 [bottom row] and Supplemental Video 4). The patient was discharged the next day and is under close ambulatory surveillance.

## Discussion

CNs have been reported in 2% to 8% of culprit lesions^1^, ^2^, ^3^ and are associated with older age (>60 years), renal dysfunction, diabetes, coronary artery bypass grafting surgery, and extensive calcification, with similar prevalence in men and women.^3^^,^^4^ CNs develop mainly in vascular segments exposed to bending stress, such as proximal-to-mid segments of a highly calcified tortuous right coronary artery and the bifurcation of the LMCA,^4^ and are associated with increased rates of stent failure.^5^ While noneruptive CNs usually affect stent expansion and result in eccentric stent configuration, eruptive CNs yield more easily, resulting in greater and more symmetric stent expansion. Nonetheless, eruptive CNs have target lesion revascularization rates twice as high as noneruptive CNs, usually occurring earlier within the first year,^6^ with reports as early as 2 weeks post-PCI.^7^

Two recurrent episodes of CN reprotrusion between 2 layers of stents within 1 year, coupled with the intravascular ultrasound and OCT images of calcium fragment protrusions with irregular luminal surfaces, indicate that an eruptive CN was the most likely mechanism of stent failure in our case. We cannot determine whether the in-stent CN protrusion was associated with the underlying plaque type before the first stent implantation or developed over time, accompanying calcified neoatherosclerosis.

The optimal management of an in-stent protrusion of an eruptive CN is unknown, in large part because the biology of coronary eruptive CN and response to angioplasty is poorly understood. One pathological study suggests that fibrous cap disruption in CN with overlying thrombosis is initiated through the fragmentation of necrotic core calcifications, stimulating further growth.^4^ Interestingly, during the period of recurrent CN protrusions into the LCx stent, the untouched LMCA CN remained stable. Therefore, we speculate that the repetitive and aggressive fragmentations we caused in the CN at the LCx ostium could reinitiate the vascular healing process after each procedure, stimulating the repeated protrusions of the CN. Our patient failed virtually all modalities of calcium modification as well as 2 layers of DES. It seemed unlikely that a third stent layer would succeed, leading to our efforts to enlarge the lumen with aggressive plaque modification techniques without implanting another stent. We hoped that brachytherapy would interfere with the inflammatory, active biology that promoted the eruptive CN regrowth, but there is little evidence of an effect. Similarly, it seems unlikely that local drug delivery with drug-eluting balloons in a heavily calcified and active plaque would be effective. It is uncertain whether a less aggressive in-stent CN manipulation would be more likely to sustain the final procedure result.

We are early in our understanding of this clinical entity, yet we are faced with the clinical need to manage the patient with in-stent CN without a reliable strategy. For now, we find ourselves sailing in dark waters and scraping the tip of the iceberg.

## Peer Review Statement

Section Editor Daniel Chamié had no involvement in the peer review of this article and has no access to information regarding its peer review. Full responsibility for the editorial process for this article was delegated to Deputy Editor Suzanne J. Baron.
